# Electrospun Polyurethane-Based Nanofibrous Membranes Functionalized with UiO-66-NH_2_ for Water Remediation

**DOI:** 10.3390/polym18091065

**Published:** 2026-04-28

**Authors:** Peio Martinez, Roberto Fernández de Luis, Jorge Sáiz, José Manuel Laza, Hugo Salazar, Alazne Gutiérrez, Rosa M. Alonso, José Andrés Fernández, Senentxu Lanceros-Mendez, Antonio Veloso-Fernández

**Affiliations:** 1 BCMaterials, Basque Center for Materials, Applications and Nanostructures, UPV/EHU Science Park, 48940 Leioa, Spain; peio.martinez@bcmaterials.net (P.M.); roberto.fernandez@bcmaterials.net (R.F.d.L.); jorge.saiz@bcmaterials.net (J.S.); hugo.salazar@bcmaterials.net (H.S.); senentxu.lanceros@bcmaterials.net (S.L.-M.); 2 Department of Physical Chemistry, Faculty of Science and Technology, University of the Basque Country UPV/EHU, Barrio de Sarriena, s/n, 48940 Leioa, Spain; josemanuel.laza@ehu.eus (J.M.L.); josea.fernandez@ehu.eus (J.A.F.); 3 Department of Chemical Engineering, Faculty of Science and Technology, University of the Basque Country UPV/EHU, Barrio de Sarriena, s/n, 48940 Leioa, Spain; alazne.gutierrez@ehu.eus; 4 FARMARTEM Group, Department of Analytical Chemistry, Faculty of Science and Technology, University of the Basque Country UPV/EHU, Barrio de Sarriena, s/n, 48940 Leioa, Spain; rosamaria.alonso@ehu.eus; 5 IKERBASQUE, Basque Foundation for Science, 48009 Bilbao, Spain

**Keywords:** polyurethane, electrospinning, water remediation, adsorption, MOF, methylene blue

## Abstract

Water contamination resulting from anthropogenic activities poses a critical threat to ecosystems and human health. The development of efficient, sustainable, and selective materials for water purification has therefore become a pressing necessity. In this study, polyurethanes (PUs) with tailored soft and hard segments were synthesized and characterized to evaluate their suitability for the fabrication of electrospun membranes. ATR-FTIR confirmed successful polymerization, while thermal analyses revealed that molecular design strongly influences the polymers’ thermal behavior. Among the synthesized materials, only two PUs exhibited solubility and spinnability, leading to homogeneous nanofibrous mats with average fiber diameters of approximately 500 nm. To enhance the adsorption capacity, specific surface area and interaction diversity of the membranes, metal–organic framework (MOF) particles were incorporated into the polymer solutions prior to electrospinning, allowing their immobilization within the fibrous polymer matrix. The resulting hybrid membranes showed remarkable improvements in methylene blue uptake, increasing from 29 to 34 mg·m^−2^ in pristine membranes and 57 to 115 mg·m^−2^ in the MOF-containing ones. This enhancement was attributed to the synergistic effect between the aromatic urethane structures and the MOF linkers, as well as to the increased effective surface area provided by the nanofibrous architecture. The results demonstrate the potential of electrospun PU-based membranes as pollutant removal, combining structural versatility, functional tunability, and compatibility.

## 1. Introduction

Clean water is an essential resource for industrial use and biological needs, which is being polluted due to increasing anthropogenic activity, making its scarcity one of the most pressing challenges of the 21st century [1,2]. This has led to the accumulation and discharge of hazardous chemicals like heavy metals and pharmaceuticals, putting ecosystems and human health at risk [3,4]. The nature of these products is wide and difficult to categorize; however, they are often persistent and toxic. For example, from the textile industry, synthetic dyes pose a threat as they can change water quality even at low concentrations and can also hinder light penetration in aquatic environments [5,6].

Although pollutants can be removed from water sources by a handful of conventional methods (coagulation–flocculation and oxidation), they have limitations concerning selectivity and operational costs [7,8]. In comparative terms, adsorption is the most suitable alternative due to its superior efficiency, simple operation, cost-effectiveness, and lack of sludge generation [9]. Sorbent materials should be highly efficient, easy to process and apply, do not induce the generation of secondary chemicals, and have a low cost. A wide variety of metal oxides, carbon-based, zeolites, and, more recently, metal–organic framework (MOF) materials have been applied to efficiently adsorb pollutants of different natures and chemistries from real water matrices [10]. Nevertheless, the main limitation relies on their powdered nature, which hinders their real applicability since their recovery and reutilization are time- and energy-consuming.

A common alternative strategy is to employ membranes from synthetic materials to support these powdered sorbents and selectively adsorb or reject pollutants from an effluent of water, offering versatility and continuous operation depending on their porosity, surface chemistry, and functionalization [11,12]. Furthermore, the pristine membrane itself is an essential aspect of the adsorption process, making its composition and processing fundamental choices. The processing of the material is the main force for pore generation, setting their characteristics [13]. Pores are one of the most important factors for water treatment membranes; not only do they define the specific area but they also affect the tortuosity of the diffusion path, altering the efficiency of the adsorption. Size exclusion and pore connectivity are the involved mechanisms through which processing modifies adsorption; size exclusion restricts pollutants bigger than the pore diameter from traversing the membrane, while pore connectivity lengthens the effective path of the effluent through the membrane. Increasing the distance of the effluent within the membrane enhances the interactions provided by the material’s functional groups.

Chemical composition is the main property modulating the specificity of the membrane, serving several purposes from pore characterization to membrane application. One of the methods used to map pores and their chemical environment involves fluorescent probing, attaching fluorescent molecules to the inner walls of pores whose changes are monitored through microscopy [14]. These probes must have high adherence to pore walls, limited by the interactions provided by functional groups present in the pores, which are also responsible for adsorbing pollutants. The most well-known interactions are electrostatic interactions (which can be modulated by medium pH or ionic strength to variate specificity, even simulating the biased transport of ionic species in cells [15,16]), hydrogen bonding and π-π interactions, which can all be considered when designing polymeric membranes. Each individual interaction caters to a wide array of pollutants that share the same kind of interaction, while a careful combination of them tailors for highly specific adsorption processes. This way, a synergistic approach can be achieved when combining adsorbent powders with polymeric membranes.

Membranes can be obtained from several processes, depending on the requirements of their application and the processability of the specific materials [17,18]. In the case of wastewater treatment, high interconnected porosity is a desirable characteristic that increases the surface of the membrane, thus improving the availability of chemical groups that act as active sites for adsorption. One way to obtain such characteristics is through electrospinning, a technique that applies high voltage to a polymeric solution, causing it to stretch and solidify by evaporating the solvent before it reaches a collector. This process creates porous nanofibrous mats with high surface-to-volume ratios, which highly enhances permeability and adsorption capacity [19,20,21]. Furthermore, electrospinning allows the direct incorporation of functional fillers—such as nanoparticles or MOFs—within the polymer matrix, expanding the range of interactions between the membrane and the pollutants [22,23].

Polymeric materials have the potential to be modified during their synthesis and processing, making them ideal due to their adaptability and ease of functionalization. One of these polymeric materials are polyurethanes (PUs), which are usually synthesized from three precursors that determine their properties. These properties are determined by the proportions of hard and soft segments in the material, which are defined by the precursors used. The soft segment is typically comprised by the polyol precursor, while the hard segment is usually comprised by the diisocyanate and the short chain diols [24,25,26]. Changing these components offers a wide range of characteristics and, therefore, potential applications tailoring their elasticity, hydrophobicity, and reusability. These polymers also offer the possibility to use reusable bio-based components like polycaprolactone (PCL), poly(tetramethylene ether) glycol (PTMG) or several low-molecular-weight diols, reducing dependency on fossil resources [27,28].

In this work, a series of polyurethanes (PUs) were synthesized using different combinations of polyols, diisocyanates, and chain extenders to explore how molecular design influences the resulting material properties. After synthesis and preliminary screening, only two PU formulations were suitable for electrospinning, enabling the fabrication of nanofibrous membranes with high surface area and interconnected porosity, features that are particularly advantageous for water purification applications.

To further enhance adsorption performance and provide deeper versatility from synthetic design, UiO-66-NH_2_, a zirconium-based photocatalytic metal–organic framework (MOF), known for its high surface area, tunable porosity, and chemical stability, was incorporated into the electrospun fibers [29]. This choice incorporates a higher number of π-π interactions and hydrogen bonds that can be designed from the synthetic step, already present in PU chemistry, while increasing their availability through increased specific surface area. This addition should synergize with the PU chemistry by enhancing its ability to adsorb the target dye, as well as other possible pollutants. This hybrid approach leverages both the mechanical flexibility and processability of PUs and the strong adsorptive capacity of MOFs, while potentially mitigating potential environmental dispersion owing to physical confinement within the polymeric matrix. As a result, the developed membranes exhibit improved capacity for the removal of model contaminants such as methylene blue, a synthetic dye used in textile industry.

The present study aims to demonstrate the feasibility of integrating polyurethane chemistry, MOF functionalization and electrospinning technology to produce high-performance membranes for water remediation. By combining efficient adsorption with a safer and more sustainable strategy for deploying nanomaterials, this work contributes to the development of advanced filtration platforms that balance performance, processability and environmental responsibility.

## 2. Materials and Methods

### 2.1. Reagents

For polyurethanes synthesis, all the reagents were provided by Sigma Aldrich (St. Louis, MO, USA). Two polyols with the same M_w_ = 2000 g/mol were utilized: polycaprolactone (PCL2000) and poly(tetramethylene ether) glycol (PTMG2000). As diisocyanates, aromatic 4,4′-diphenylmethane diisocyanate (MDI) and aliphatic 1,6-hexamethylene diisocyanate were used, while two diols acted as chain extenders (CE), 1,4-butanediol (BDO) and ethylene glycol (EG). As solvent N,N’-dimethylformamide (DMF, 99.9%) was used.

For MOF UiO-66-NH_2_ synthesis, zirconium chloride (ZrCl_4_, 98%) and 2-aminoterephthalic acid (BDC-NH_2_, 99%) were purchased from Alfa Aesar (Ward Hill, MA, USA), while methanol (99%) was used for its washing, obtained from Labkem (Barcelona, Spain).

### 2.2. Synthesis of Polyurethanes

**Figure 1:**
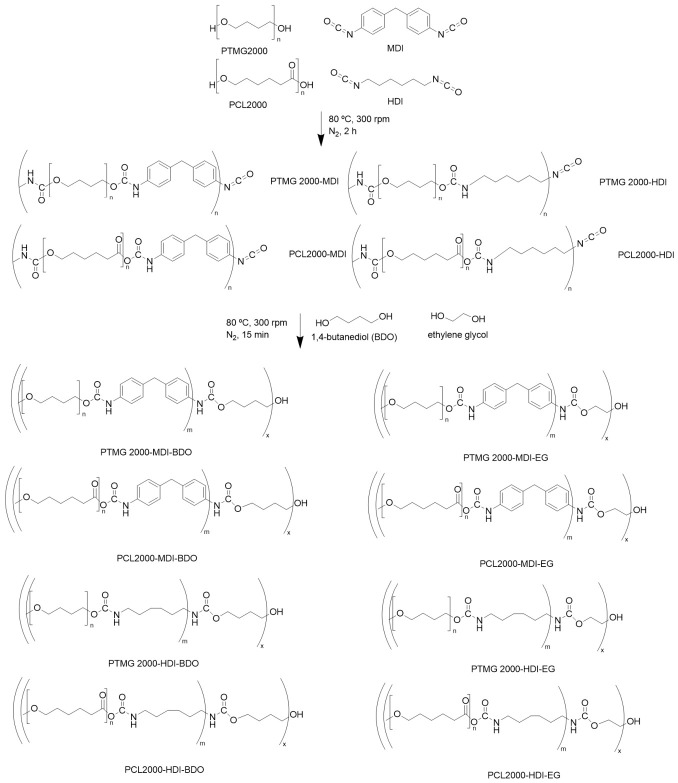
Scheme of the PU synthesis by prepolymer method [30,31].

A two-step solvent-free procedure (prepolymer method) was employed for the synthesis of the polyurethanes (Figure 1), keeping an inert nitrogen atmosphere and a stirring velocity of 300 rpm throughout the process. The prepolymer was achieved in the first step, where the polyol was added to a five-necked flask and heated to 80 °C until molten, then, the diisocyanate was added and the mixture was stirred for 2 h at 80 °C. After that, the CE was added, and the reaction went on for 15 min at 80 °C. Finally, the mixture was poured into a Teflon covered steel mold and pressed at 100 °C and 25 bar for 24 h.

Proportion of materials followed a stochiometric ratio of N = 2, defined by polyol_1_:diisocyanate_N+1_:CE_N_. In this way, a polyurethane of molar composition polyol_1_:diisocyanate_3_:CE_2_ and isocyanate/hydroxyl ratio of 1 was achieved, with the objective of minimizing unreacted isocyanate groups. Naming of samples followed this same scheme; for example, a polymer composed of PTMG as polyol, MDI as diisocyanate and BDO as CE will be named PTMG2000-MDI-BDO.

### 2.3. UiO-66-NH_2_ MOF Synthesis

A solvothermal method was employed for the synthesis of UiO-66-NH_2_. A total of 0.5418 g of ZrCl_4_ were dissolved in 60 mL of DMF; in parallel, 0.185 g of 2-aminoterephthalic acid (BDC-NH_2_) were suspended in 1.5 mL of distilled water. Once both solutions were homogeneous, the BDC-NH_2_ suspension was added dropwise to the ZrCl_4_ solution, then the mixture was poured in a Teflon lined autoclave that was kept at 80 °C for 24 h. Once the reaction ended, the solid was retrieved by centrifugation and washed thrice with 100 mL of DMF and 100 mL of methanol over three days. Finally, the product was dried at 80 °C for one day [29].

### 2.4. Attenuated Total Reflectance Fourier Transform Infrared Spectroscopy (ATR-FTIR)

A Nicolet Nexus 670 FT-IR (Thermo Fisher Scientific Inc., Madison, WI, USA) spectrophotometer equipped with a zinc selenide (ZnSe) crystal-based ATR accessory was used to characterize the functional groups in the polymers and conversion rate (CR%) of the isocyanate groups, which become urethane groups after their reaction with hydroxyl groups present in both polyol and CE. This leads to a decrease in the characteristic signal of isocyanate asymmetric stretching around 2275 cm^−1^ [32], which can be quantified to determine the percentage of isocyanate that has reacted by Equation (1).

(1) $$CR\left(\%\right)=(1-\frac{A_{P}}{A_{R}})\cdot 100$$

where *A_p_* is the absorbance of the polyurethane and *A_R_* is the absorbance of the diisocyanate reactive, both at their normalized (relative to the CH_2_ vibration peaks) isocyanate peak in 2275 cm^−1^ [33]. The scans were taken with 4 cm^−1^ of resolution from 4000 to 650 cm^−1^, with a total of 32 scans per sample.

### 2.5. Differential Scanning Calorimetry (DSC)

Thermal transitions were studied in a DSC Mettler Toledo 822 instrument (Greifensee, Switzerland). Samples, 20–25 mg in aluminum pans, were subjected to two heating and cooling scans from −100 °C to 200 °C at 20 °C·min^−1^ under nitrogen flux of 20 mL·min^−1^.

### 2.6. Thermogravimetric Analysis (TGA)

Degradation of materials was determined by thermogravimetric analysis (TGA) temperature-programmed oxidation (TPO) in a TA Instruments TGA Q5000 thermobalance (New Castle, DE, USA) by using alumina pans. The sample was stabilized at 25 °C with an air stream (50 mL min^−1^) for 5 min and then the combustion step is carried out, heating up with a ramp of 15 °C min^−1^ and keeping it constant at 600 °C for 5 min.

### 2.7. Viscosity Measurements

A programmable Brookfield DV2TRV rotational viscometer (Middleboro, MA, USA) was employed to measure viscosity within the range of 100 to 40 million centipoise (cP). This instrument is capable of measuring density at various temperatures as well. It is equipped with multiple spindles to accommodate different precision and speed requirements. In this study, the SC4-21 spindle for low-volume samples was used and viscosities were measured thrice.

### 2.8. Electrospinning Process

The organic solvents THF and DMF were screened to evaluate the solubility of the synthesized polyurethanes in the 25–50 °C temperature range. Solubility tests were conducted to identify formulations compatible with solution processing and electrospinning, enabling the selection of candidates suitable for further fabrication and characterization. Of all 8 synthesized samples, two showed appropriate solubility for electrospinning (PTMG2000-MDI-BDO and PCL2000-MDI-EG) in the tested conditions, DMF being the only suitable solvent.

**Figure 2:**
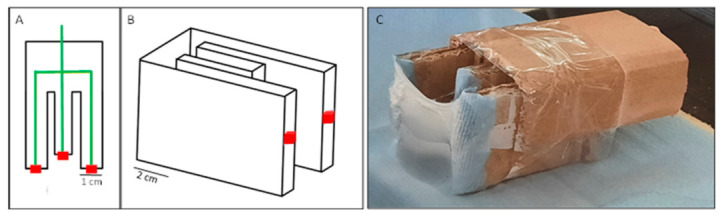
Prototype diagram of the homemade collector system: (**A**) top view, (**B**) side view highlighting the internal wiring in green and the metal contacts in red; (**C**) photograph of the cardboard prototype collector system holding a freshly synthesized PU membrane.

The voltage was applied with a HiTek Power OL400/303PD power supply; using a Syringepump NE-300 syringe pump, a constant polymer solution flow rate was provided through the syringe needle. Fibers were collected on a homemade collecting plate at varying distance from the needle, consisting of a U-shaped wooden structure with two parallel vertical metallic plates at 5 cm separation, covered in aluminum foil (Figure 2). This setup provided a simple retrieval method for all samples, as the membrane grew in the empty space between the plates instead of adhering to the foil. For all trials, temperature was kept at 20 ± 3 °C and relative humidity at 50 ± 2%.

For both samples, polymer solutions at 10% *m*/*v* (10 g of polymer per 100 milliliters of DMF) were prepared by stirring at 50 °C for 24 h. After reaching room temperature, the solutions were placed in a 5 mL Terumo plastic syringe fitted with a steel needle. In the case of MOF addition, 5 g per 100 g polymer mass (5% m/m concentration) were added to the solution once it reached room temperature, stirring for additional 24 h before placing the solution in the syringe. Higher MOF load resulted in needle clogging and particle precipitation, causing loss of material and unstable electrospinning.

The volume of solution in the syringe was typically 5 mL. A minimum runtime of 180 min was used (total volume of 1.8 mL, obtaining 70 ± 10 mg of sample) to produce membranes. The collected samples were dried at room temperature for characterization. Operational parameters were systematically optimized by varying applied voltage (10–20 kV), flow rate (0.3–1.5 mL h^−1^), and tip-to-collector distance (7.5–20 cm). Optimal fiber formation was achieved at 12 kV, 0.75 mL h^−1^, 20 cm distance, and 18 G needle diameter for PTMG2000-MDI-BDO. In the case of PCL2000-MDI-EG 20 kV, 0.9 mL h^−1^ 20 cm distance and 18 G needle diameter were used.

### 2.9. Scanning Electron Microscopy (SEM)

Scanning electron microscopy (SEM) was employed to capture images of the fibers obtained through electrospinning utilizing the HITACHI S-4800 model (Tokyo, Japan). To enhance the resolution of the samples, it was necessary to increase their electrical conductivity by coating them with a nanometric gold layer using the EMITECH K550X sputtering tool. Finally, the average diameter and standard deviation of the fibers were determined by ImageJ software [34] version 17.5.30, measuring 20 random fibers in each micrograph.

### 2.10. Methylene Blue (MB) Adsorption

In order to test the use of these films as dye adsorbents in water purification applications, an aqueous solution of MB (9 mg/L) was prepared as a model of polluted wastewater while avoiding membrane saturation [35,36,37]. This choice was driven by its extended use in the textile industry due to its high adhesion to cotton fibers, producing high amounts of dye-polluted wastewater that pose a threat to aquatic and human life [38]. Samples of different self-standing membranes with a surface area of 0.6 ± 0.1 cm^2^ and weight of 2.0 ± 0.5 mg were introduced in vials containing 4 mL of the prepared MB solution. Surface was calculated geometrically by cutting rectangular samples of the electrospun membranes, which were then weighed and measured. Considering the low weight of the materials, surface was used to normalize adsorption capacity as a more reliable parameter. After adsorption time, the remaining dye in each solution was quantified by UV–VIS spectroscopy. Each solution absorbance at 663 nm was monitored periodically until equilibrium was reached (0.5, 1, 2 and 3 h) with a Cintra 303 UV–Visible Spectrophotometer (GBC Scientific Equipment Ltd., Melbourne, Australia), employing a corresponding standard curve for MB dissolved in MilliQ water (Figure S2). Adsorption capacity was calculated by using Equation (2):

(2) $$A=\;\frac{C_{0}-C_{t}}{S}\cdot V$$

where *A* is the adsorption capacity (mg·m^−2^), *C*_0_ and *C_t_* are the initial and equilibrium MB concentration, respectively, *S* is the geometrically measured sample area (m^2^) and *V* is the used MB solution volume (L).

## 3. Results and Discussion

### 3.1. Synthesis and Chemical Characterization of Polyurethanes

Different PUs were synthesized by combining two types of polyols with the same molecular weight 2000 g·mol^−1^—poly(tetramethylene ether) glycol (PTMG2000) and polycaprolactone diol (PCL2000)—with two different CEs, 1,4-butanediol (BDO) and ethylene glycol (EG), using 4,4′-methylenediphenyl diisocyanate (MDI) or 1,6-hexamethylene diisocyanate (HDI) as the diisocyanate component. Solubility screening revealed that only two out of the eight synthesized polyurethanes displayed adequate solubility in DMF, allowing successful processing by electrospinning. The remaining formulations showed insufficient dissolution or poor processability in the tested solvents. Based on these findings, subsequent analyses and in-depth characterization were restricted to the two polymers exhibiting optimal solubility and spinnability, as they demonstrated the most promising characteristics for membrane fabrication.

ATR-FTIR was used to characterize the functional groups present in the synthesized PUs, as well as to determine the CR of the isocyanate groups (Figure S1). Firstly, there is a scarce presence of -OH groups in the 3600–3400 cm^−1^ range, indicating the complete reaction of the polyol and CE with isocyanate groups, barring the terminal groups of polymeric chains. Additionally, the characteristic stretching vibrations of the amino group (-NH) are present at 3250–3400 cm^−1^ and in-plane bending double peak at approximately 1612 and 1600 cm^−1^, corresponding to the flexion vibration of the -NH group in the plane [26]. The carbonyl (-C=O) signal is also present as a double peak around 1710 cm^−1^, confirming both amide (1700 cm^−1^) and ester (1730 cm^−1^) presence, signaling the appearance of the urethane group [39].

Further, the amide II band (N-H bending and C-N stretching) is detected at 1530 cm^−1^. Other signals that are identified are the ones found at 1410 and 1450 cm^−1^, attributed to the asymmetric and symmetric bending of the CH_2_ group, respectively. Finally, different vibrations of the CH_2_ group can be seen at 2940–2920 and 2860–2840 cm^−1^, corresponding to the asymmetric and symmetric stretching [40]; the vibration of the bond CH_2_-O is mainly found for the PTMG samples at 2796 cm^−1^, due to the absence of carbonyl groups in the polyol chain [40]. The complete description of these signals can be found in Table S1.

Residual isocyanate groups were identified by the band at 2260–2280 cm^−1^, allowing the estimation of CR by applying Equation (1). The PTMG2000-MDI-BDO formulation achieved the highest CR (≈90%), while PCL2000-MDI-EG exhibited the lowest (≈64%), suggesting incomplete reaction possibly due to steric hindrance or reduced miscibility between components.

### 3.2. Electrospinning and Fiber Morphology

**Figure 3:**
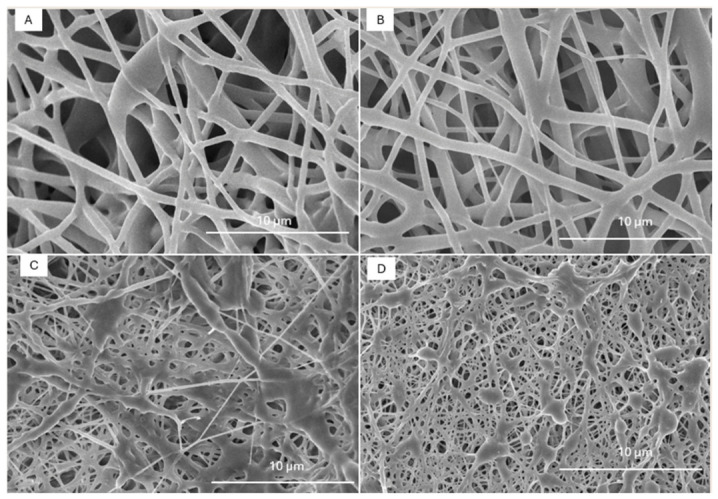
SEM micrographs of electrospun nanofibers for samples: (**A**) PTMG2000-MDI-BDO; (**B**) PCL2000-MDI-EG; (**C**) PTMG2000-MDI-BDO with 5% MOF; and (**D**) PCL2000-MDI-EG with 5% MOF.

The polymers selected, PTMG2000-MDI-BDO and PCL2000-MDI-EG, were processed by electrospinning. SEM images (Figure 3A,B) revealed continuous and bead-free fibers with average diameters of 500 ± 107 nm for PTMG2000-MDI-BDO and 513 ± 150 nm for PCL2000-MDI-EG. The relatively broad diameter distributions were attributed to the low viscosity of the polymer solutions (≈420–470 cP), leading to limited chain entanglement and occasional jet instabilities. Despite this, both materials produced coherent nanofibrous mats suitable for further functionalization and adsorption studies.

Optimal fiber formation was achieved at 12 kV, 0.75 mL h^−1^, 20 cm distance and 18 G needle diameter for PTMG2000-MDI-BDO. In the case of PCL2000-MDI-EG 20 kV, 0.9 mL h^−1^ 20 cm distance and 18 G needle diameter were used. The difference may arise due to the stiffness of the polymeric chains. The higher stiffness in both hard and soft segments for the PCL-based sample hinders chain alignment, needing a higher voltage to allow proper fiber formation, despite the similar viscosity of both polymer solutions. This voltage implied the use of a higher polymer flux to supply sufficient material for constant uninterrupted nanofiber processing, which could explain the slightly higher diameter distribution in this sample. High needle-to-collector distance was needed in both cases to avoid solvent reaching the nanofibrous mat.

The addition of UiO-66-NH_2_ particles affected the morphology of the nanofibrous mats (Figure 3C,D), as well as a small change in processing parameters. The applied voltage had to be increased in both cases due to the added interactions, which made the chains more difficult to align during fiber formation. The voltages needed to be changed to 14 kV for the PTMG-based polymer and to 21 kV for the PCL-based one (Figure S3 shows micrographs of the pristine membranes at different voltages, showing a lower relation between voltage and diameter than the stretching attributed to MOF inclusion). In addition to the higher voltage causing superior fiber stretching, the MOF particles act as nodes where polymeric mass attaches, strongly reducing average fiber diameter for both polymers. In the case of PTMG2000-MDI-BDO, fiber diameter reduced to 354 ± 139 nm, while PCL2000-MDI-EG reduced to 276 ± 93 nm. The drastic change for the PCL sample could be attributed to its superior phase mixing, having less interactions with the MOF particles and generating a higher number of homogeneously distributed nodes that stretch the fibers between them. For the PTMG samples, due to its lower phase mixing, it generates a higher amount of hydrogen bonds with the MOF, hindering its dispersion and generating less nodes with higher MOF loading. Further trials with higher MOF content resulted in loss of filler due to its precipitation and needle clogging, possibly due to lack of proper particle dispersity. Despite the higher apparent surface area, specific BET measurements could not be performed due to the low mass of the membranes, making them unreliable.

### 3.3. Thermal Characterization

**Figure 4:**
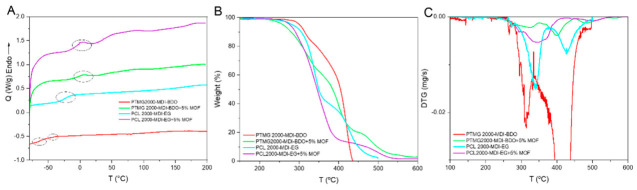
DSC (**A**), TGA (**B**) and DTG (**C**) thermograms of the synthesized PUs. PTMG2000-MDI-BDO in red, PTMG2000-MDI-BDO + 5% MOF in green, PCL2000-MDI-EG in cyan, and PCL2000-MDI-EG+5% MOF in purple.

With the objective of understanding the polymers’ thermal properties, DSC was used. Figure 4A shows that the electrospun PUs glass transition temperatures (T_g_, onset method, crossing point of the tangential lines extended from the beginning of the slope change) below 0 °C, confirming their elastomeric nature at room temperature. PTMG2000-MDI-BDO (red line) displays two distinct glass transitions at −65 °C and at −42 °C (Enhanced thermogram in Figure S4), which could be attributed to the T_g_ of the soft and hard segment domains, respectively, indicating separated phases. In the case of PCL2000-MDI-EG (cyan line), it presents a single T_g_ at −24 °C. The absence of melting peaks confirmed that all systems are amorphous, with no crystalline domains. The higher T_g_ observed for the PCL-based formulation suggests a stiffer soft segment and stronger intermolecular interactions compared to PTMG-based analog, possibly attributed to the stronger hydrogen bonding present between the PCL-based chains and the higher steric hindrance of the carbonyl groups it possesses [41].

As can be seen in the thermograms, the inclusion of MOF particles erases the presence of glass transition temperatures while creating small endothermic transitions around 0 and 100 °C. The absence of glass transitions can be attributed to the PU-MOF interaction, inhibiting chain movement and delaying glass transition until overlapping with the endothermic transitions, complicating the identification of apparent T_g_s. The endothermic transitions could be attributed to water melting and evaporation trapped in the MOF structure due to its hydrophilic nature.

These results demonstrate that both the nature of the polyol and the CE have a pronounced effect on the polymer microphase structure. Shorter CE (EG) and stiffer polyol (PCL) increase T_g_, reducing segmental mobility and flexibility [32]. At the same time, adding MOF particles seems to reduce chain mobility due to added interactions, having greater effect on PTMG samples due to lower phase mixing that allows for greater interaction with the particles.

In order to understand the thermal degradation of the PU samples, their TGA and DTG were evaluated (Figure 4B,C). All samples are thermally stable below 250 °C, having a first degradation step at around 300–400 °C, where the decomposition of PU hard segment occurs. A second major degradation step is found at 350–450 °C, where the polyol breaks down, that is, the soft segment of PU [42]. Specifically, in the case of PTMG2000-MDI-BDO these steps appear at 313 °C (PTMG degradation) and 420 °C (BDO and MDI degradation), while PCL2000-MDI-EG shows slightly delayed first and second steps at 338 °C (PCL degradation) and 427 °C (EG and MDI degradation). This could also be attributed to the stronger intermolecular interactions present in PCL-based PUs, making them more thermally stable, as also seen by DSC [32].

Furthermore, the thermal degradation of the electrospun samples containing MOF shows that degradations corresponding to the MOF occur in two distinct steps. The first one is at 350 °C, attributed to the breakdown of the organic linker (BDC-NH_2_), while the second one is observed around 470–500 °C attributed to the total loss of the structure (remains of Zr clusters) [43]. Both in PTMG2000-MDI-BDO and PCL2000-MDI-EG, the inclusion of these particles generates a shift in the decomposition of the different segments: the degradation of the hard segment is delayed while the degradation of the soft segment is accelerated.

The effect is major in the PTMG sample, where the hard segment degradation shifts from 313 °C to 331 °C and the soft segment degradation shifts from 420 °C to 372 °C. The impact of the MOF particles is less noticeable in the PCL sample, where the hard segment degradation shifts from 338 °C to 342 °C and the soft segment degradation shifts from 409 °C to 400 °C. This phenomenon could be attributed to the specific PU-UiO-66-NH_2_ interactions and the difference in phase mixing. The MOFs aromatic groups and carboxylates interact with the aromatic MDI and the hard segments’ urethane groups, increasing their stability while restricting the intermolecular hydrogen bonding that stabilizes the soft segments. This is more noticeable in the PTMG sample due to lower phase mixing in comparison to the PCL sample, as seen by the T_g_s of the materials, which makes interaction with the hard phase more accessible, impacting with greater effect on the differentiated phases of the PTMG sample [41,44].

### 3.4. Methylene Blue Adsorption

**Figure 5:**
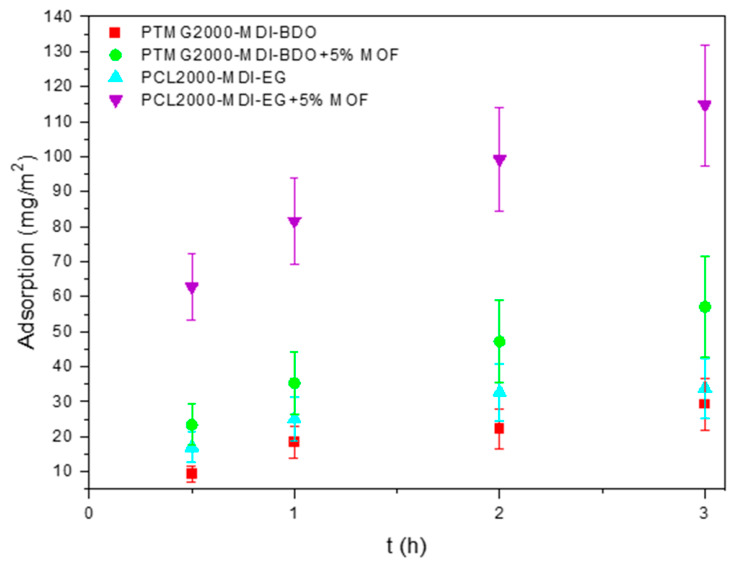
Adsorption of MB by the electrospun membranes: pristine PTMG2000-MDI-BDO (red), PTMG2000-MDI-BDO + 5% MOF (green), pristine PCL2000-MDI-EG (cyan) and PCL2000-MDI-EG + 5% MOF (purple).

The adsorption performance of the electrospun membranes was evaluated using methylene blue (MB) as a model organic dye (Figure 5). Both pristine PU membranes exhibited moderate adsorption capacities, reaching 29 mg·m^−2^ for PTMG2000-MDI-BDO and 34 mg·m^−2^ for PCL2000-MDI-EG. The adsorption mechanism was mainly driven by weak π–π interactions between the aromatic rings of the MDI moiety in the PU and the MB dye molecules. By comparing both polymeric matrices, PCL2000-MDI-EG showed higher MB adsorption when compared to PTMG2000-MDI-BDO due to their different chemical functionalities and polarities. PCL is composed by ester carbonyl (–C=O) groups along its backbone, which has a higher dipole moment than the ether (–C–O–C–) linkages in PTMG. The carbonyl groups act as hydrogen-bond acceptors and enable dipole–dipole interactions with the cationic MB dye. Also, the higher polarity of PCL promotes electrostatic interactions with MB, whereas PTMG provides weaker interaction sites [45].

To enhance adsorption capacity and provide the membrane with further functionalization versatility, UiO-66-NH_2_ MOF particles were incorporated into the polymer solutions prior to electrospinning at 5 wt. % relative to polymer mass. The resulting hybrid membranes displayed a significant increase in dye uptake: 57 mg·m^−2^ for PTMG2000-MDI-BDO and 115 mg·m^−2^ for PCL2000-MDI-EG. This improvement could be attributed to the theoretically higher surface area of the smaller fibers, and the Zr_6_-oxo clusters and functional groups of UiO-66-NH_2_, which provide additional π–π and electrostatic interactions, together with hydrogen bonding, through the aromatic terephthalic acid linker and the –NH_2_ functionalities [46,47].

The superior performance of PCL2000-MDI-EG + UiO-66-NH_2_ may also be related to its higher rigidity, leading to better dispersion and potential retention of MOF particles within the fiber matrix [48]. These findings support the synergistic effect between the PU matrix and MOF additive, enabling efficient dye removal while maintaining the structural integrity of the membranes.

## 4. Conclusions

Despite the successful synthesis, only two of the proposed PU formulations were soluble in DMF, making them suitable for electrospinning trials. The optimized formulations—PTMG2000-MDI-BDO and PCL2000-MDI-EG—were successfully processed into uniform nanofibrous membranes with diameters around 500 nm using a homemade collector. Notably, electrospinning proved to be a highly advantageous fabrication technique, enabling the production of nanofibrous membranes while simultaneously serving as a possible strategy to immobilize MOF nanoparticles, thereby reducing the risk of nanomaterial release into the environment. This is a critical aspect, as free MOFs and other nanomaterials may pose environmental concerns if not properly confined within a stable matrix.

Furthermore, these formulations enabled the homogeneous dispersion of UiO-66-NH_2_ MOF particles within the polymer solutions, which were also successfully electrospun into finer nanofibers with diameters around 300 nm. When tested for methylene blue adsorption, both pristine and MOF-functionalized membranes exhibited significant dye uptake, demonstrating their strong potential for wastewater remediation. The incorporation of UiO-66-NH_2_ not only enhanced adsorption performance through additional interaction sites and increased surface-to-volume ratio but also influenced fiber morphology by reducing average diameter. Increasing the naturally present interactions of the PU through this MOF seems to be an effective way of manipulating its specific surface area, making the adsorption sites more accessible to interact with the dye, be it through polymers or additives interactions.

These findings highlight the benefits of hybridizing PU matrices with porous nanostructures, combining hydrophilic and hydrophobic interactions while enabling morphological and chemical tuning through filler type and content. Overall, this study demonstrates the feasibility of developing sustainable, high-performance electrospun PU membranes as a versatile platform for contaminant removal, while simultaneously providing a safer and more environmentally responsible approach to deploying nanomaterials.

Future work will focus on refining PU molecular design to further elucidate its influence on electrospinning behavior and adsorption efficiency, with particular emphasis on the superior filler stabilization and adsorption performance observed for the stiffer PCL2000-MDI-EG system; this is of special interest regarding leaching—future work should prioritize analyzing the relationship between PU chain stiffness and filler fixation. This interplay dictates the environmental impact of the utilized materials due to degradation and filler discharge, as well as its potential for reutilization without loss of efficiency or generation of secondary pollution. Fundamental work regarding pollutant concentration and interaction must also be considered a priority, as allowing us to understand the kinetic and mechanistic principles behind the process would help extend the usability of the membranes and propose a regeneration criterion for the membranes.

Additionally, the photocatalytic activity of the MOF should be studied within the membrane to ensure this property is still active and selective enough not to affect the polymers’ stability. This is a crucial step to ensure long-term membrane use or regeneration, while avoiding membrane degradation along the contamination it carries. Finally, achieving a higher membrane mass output would turn beneficial for higher production and reliable specific-surface measurements through BET analysis.

## Data Availability

The original contributions presented in this study are included in the article/Supplementary Material. Further inquiries can be directed to the corresponding author.

## Supplementary Materials

The following supporting information can be downloaded at https://www.mdpi.com/article/10.3390/polym18091065/s1. Figure S1. Transmittance FTIR spectra of the synthesized polyurethanes. PTMG2000-MDI-BDO in red and PCL2000-MDI-EG in cyan. Figure S2. Methylene blue calibration curve in water. Figure S3. SEM micrographs of electrospun nanofibers for samples: (A) PTMG2000-MDI-BDO at 20 kV with an average nanofiber diameter of 440 ± 110 nm; (B) PCL2000-MDI-EG at 12 kV with an average nanofiber diameter of 540 ± 100 nm. Figure S4. Augmented DSC thermogram of PTMG2000-MDI-BDO. Table S1. Synthesized polyurethane FTIR spectra analysis.

## Abbreviations

ATR-FTIR	attenuated total reflectance–Fourier transform infrared
BDC-NH_2_	2-aminoterephthalic acid
BDO	1,4-butanediol
CE	chain extender
CR	conversion rate
DSC	differential scanning calorimetry
EG	ethylene glycol
HDI	1,6-hexamethylene diisocyanate
MB	methylene blue
MDI	4,4′-diphenylmethane diisocyanate
MOF	metal–organic framework
PCL	polycaprolactone
PTMG	poly(tetramethylene ether) glycol
T_g_	glass transition temperature
TGA	thermo-gravimetric analysis
THF	tetrahydrofuran
ZrCl_4_	Zirconium chloride
