# Supplementary material for: Electrospun Polyurethane-Based Nanofibrous Membranes Functionalized with UiO-66-NH2 for Water Remediation
Source: Polymers (Basel). 2026 Apr 28;18(9):1065. doi: 10.3390/polym18091065 (PMC13165193; doi:10.3390/polym18091065)
Supplement: Supplementary file 1 [file polymers-18-01065-s001.zip › polymers-4201271-supplementary.pdf]

Supplementary Information

Electrospun Polyurethane-based Nanofibrous Membranes Functionalized with UiO-66-NH_2 for Water Remediation

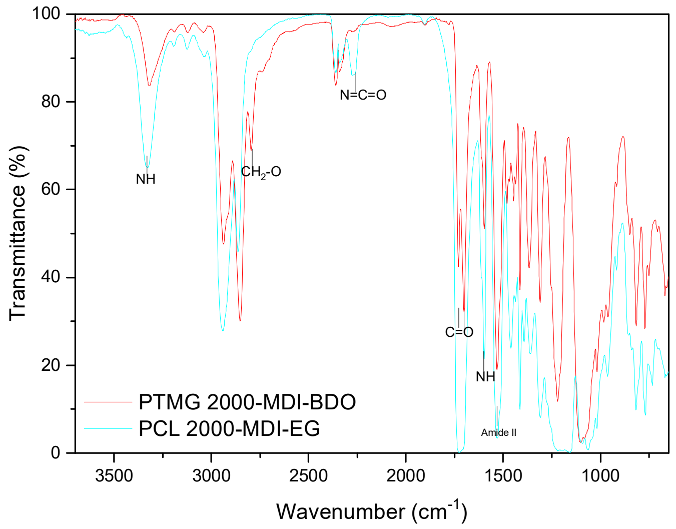

Figure S1. Transmittance FTIR spectra of the synthesized polyurethanes. PTMG2000-MDI-BDO in red and PCL2000-MDI-EG in cyan.

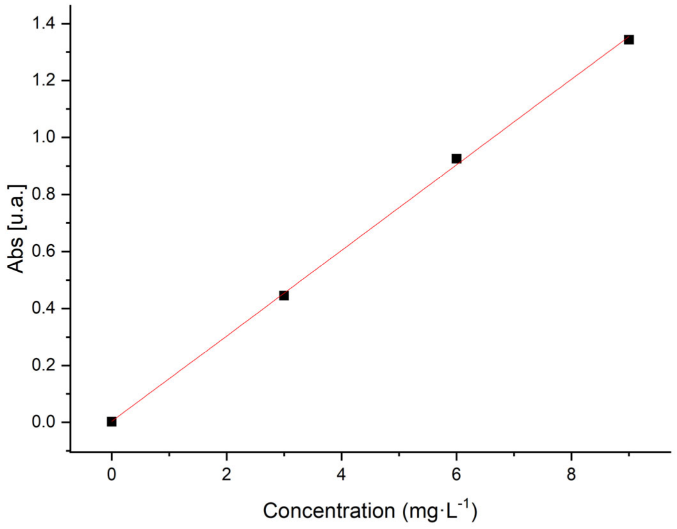

Figure S2. Methylene blue calibration curve in water.

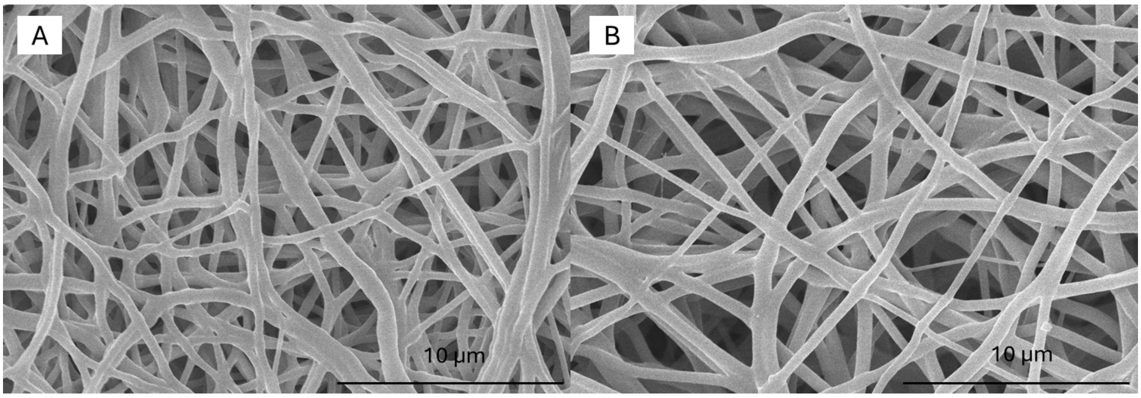

Figure S3. SEM micrographs of electrospun nanofibers for samples: (A) PTMG2000-MDI-BDO at 20 kV with an average nanofiber diameter of 440 ± 110 nm; (B) PCL2000-MDI-EG at 12 kV with an average nanofiber diameter of 540 ± 100 nm.

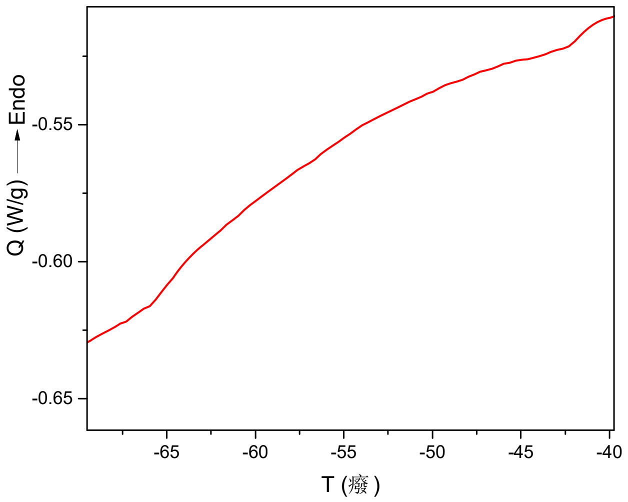

Figure S4. Augmented DSC thermogram of PTMG2000-MDI-BDO.

Table S1. Synthesized polyurethane FTIR spectra analysis.

PTMG2000-MDI-BDO	PCL2000-MDI-EG	Wavenumber (cm^{-1})	Description
-	-	3400-3600	OH
3321	3328	3250-3400	ν (N-H)
2940	2942	2940-2920	ν_{as} (CH_2)
2852	2863	2860-2840	ν_s (CH_2)
2275	2275	2260-2280	ν_{as}(N=C=O)
1730	1732	1730	ν (OC=O)
1700	1704	1700	ν (HNC=O)
1612, 1597	1612, 1597	1610-1590	δ (NH) double peak
1531	1530	1530	Amide II ($\delta_{N\text{-}H}$ + $\nu_{C\text{-}N}$)
1468	1462	1445-1485	δ_s (CH_2)
1414	1414	1414	δ_{sa} (CH_2)
2794	2796 (very weak)	2795	ν (CH_2-O)
